# Post-operative analysis of pediatric esotropia associated with high hypermetropia

**DOI:** 10.1186/s12886-019-1149-3

**Published:** 2019-07-01

**Authors:** Bo Li, Sapna Sharan

**Affiliations:** 1 Department of Ophthalmology, Ivey Eye Institute, Schulich School of Medicine and Dentistry, Western University, London, Ontario Canada; 2 Division of Ophthalmology, St Joseph’s Health Care London, 268 Grosvenor Street, London, ON N6A 4V2 Canada

**Keywords:** Hyperopia, BMR, Strabismus, Hypermetropia, Esotropia, Surgery

## Abstract

**Background:**

To describe clinical features, evaluation, surgical management and outcomes in children with esotropia associated with high hypermetropia.

**Methods:**

Medical records of healthy children who received strabismus surgery for accommodative esotropia with hypermetropia larger than spherical equivalence of + 4.0 diopters from 2009 to 2015, were reviewed.

**Results:**

A total of 47 patients were identified. The average age was 2.9 years old. The average spherical equivalence of cycloplegic refraction was + 6.0 diopters (D). All patients presented with large angle esotropia with spectacle correction. Average age of esotropia onset was 1.3 years. Average time between the onset of esotropia to spectacle correction was 7.2 months. Average duration between onset of constant esotropia to strabismus surgery was 28.1 months. Average duration between spectacle correction to strabismus surgery was 21.8 months. Post-operatively, 74.5% of patients achieved ocular alignment within 10 prism diopters (PD) of orthotropia. Overall, 66.0% patients developed sensory fusion. For patients who achieved surgical success, 71.4% developed sensory fusion, compared at 50.0% for patients who were over- or under-corrected (*p* = 0.18). For patients who received hyperopic spectacles within 6 months of esotropia onset, 92.3% developed sensory fusion, compared with 54.5% for patients who received hyperopic spectacles at 6 month or later after esotropia onset (*p* = 0.02).

**Conclusions:**

Strabismus surgery for esotropia with high hypermetropia has high rate of surgical success with low rate of under- or over-correction. There is a trend toward higher rate of sensory fusion for patients with surgical success. Shorter time interval between esotropia onset and receiving hyperopic spectacles is associated with higher rate of sensory fusion development.

## Background

The degree of hypermetropia is an important component and risk factor for refractive, accommodative and partially accommodative esotropia [1]. Many patients with purely refractive esotropia, which can fully be corrected with hyperopic spectacle correction, may develop partial accommodative esotropia over time [2, 3]. The management paradigm for esotropia associated with high hypermetropia starts with ophthalmic examination, cycloplegic refraction and full hyperopic spectacle correction. Traditionally, standard strabismus surgery for partial accommodative esotropia aims to correct the residual deviation after prescribing full hyperopic spectacle correction. However, standard surgery for the non-accommodative component of esotropia has resulted in high rate of under-correction. Under-correction rate of as high as 60% has being reported [4–6]. As a result, strategies such as prism adaptation and augmented surgery have been proposed and used to determine the surgical target for patient with esotropia associated with high hypermetropia [4, 6–10].

However, there still is a paucity of literature on the details of surgical and functional outcomes of strabismus surgery for esotropia associated with high hypermetropia. Not much is known about the potential factors that may influence the surgical and functional outcomes for this group of patients. The purpose of this study to review the surgical and functional outcomes of children who received bilateral medial rectus muscle recession surgery for esotropia associated with high hypermetropia and to identify potential factors that may influence outcomes.

## Methods

Medical records of children who received bilateral medial recession (BMR) surgery from 2009 to 2015 by a single pediatric ophthalmologist (SS) at the Ivey Eye Institute, Western University (London, Ontario, Canada), were retrospectively reviewed. Patient charts were manually reviewed by BL and patients who presented with hypermetropia with spherical equivalence of 4.0 diopters or larger were included in the study. The patient history, clinical evaluation, orthoptic assessment, surgical intervention and surgical outcomes were recorded. The following information was collected from patient charts: age of presentation/referral, age of esotropia onset, age of spectacles use, age at time of surgery, sex, past medical history, past ocular history, family history, cycloplegic refraction, amount of muscle recession, pre-operative and post-operative deviation at near and distance, pre-operative and post-operative stereopsis with Titmus Stereo Acuity plates and presence of fusion with Worth 4-dot testing (distance and near) and the need for re-operation. Exclusion criteria consisted of: past ocular history other than strabismus, hypermetropia less than + 4.0 D of spherical equivalence, as well as significant past medical history such as craniofacial abnormalities, identified genetic disorders, developmental delay and history of prematurity prior to 35 weeks. The amount of bilateral medical rectus recession was determined from standard surgical table based on the pre-operative measurement of the near deviation with spectacle correction [11]. Pre-operatively, prism adaptation testing was added to the pre-operative surgical planning if patient still demonstrated symptoms of diplopia with loose prisms at the measured near deviation in the clinic with post-operative diplopia testing. Pre-operative eso-deviation at near with spectacle correction was measured in all patients unless the patient was too young to be measured or if the patient did not comply despite best efforts. Surgical success was defined as post-operative averaged near and distance ocular alignment measured at the last follow-up within 10 PD of orthotropia on cover test. Surgical over-correction was defined as post-operative exotropia (consecutive exotropia) of more than 10 PD. Surgical under-correction was defined as post-operative residual esotropia of more than 10 PD. Research ethics approval was obtained from Western University Health Science Research Ethics Board - HSREB number 107068.

## Results

### Baseline patient characteristics

A total of 238 patient records were reviewed. Out of these, 191 patients were excluded based on the exclusion criteria and 47 patients who presented with esotropia and hypermetropia of + 4.0 diopters or more in spherical equivalence and received bilateral medical recession surgery were included. Detailed baseline patient characteristics is listed in Table 1. A total of 23 male and 24 female patients were included. The average age of patient at time of referral was 2.9 years old (range 0.5 to 8.0). All patients were born term except one born premature at 27 weeks of gestation. No patients had any previous or concurrent ocular history other than strabismus. Twenty-four patients (53.2%) elicited a definite family history of strabismus and/or amblyopia. The average age of esotropia onset was 1.3 years old (range was 0 to 3.8). The average age of starting spectacle correction for hypermetropia was 1.9 years old (range 0.3 to 4.0). The average spherical equivalence (SE) was found to be + 6.0 D (range + 4.0 D to + 9.8 D). Cycloplegic refraction was carried out with 1% Cyclopentolate eye drops in children 1 year age and older. 0.5% Cyclopentolate drops were used in less than 1 year old children. The average time between esotropia onset to start of spectacle correction for hypermetropia was 7.2 months (range 0 to 36). Thirteen patients received hyperopic spectacles within 6 month of esotropia onset and 33 patients received hyperopic spectacles 6 months or longer after the onset of esotropia. The average time between spectacle correction to strabismus surgery was 22 month (range 2 to 80). The average duration between the onset of constant esotropia to strabismus surgery was 28.1 month (range 1–82 month). The average age of patients at the time of surgery was 3.6 years old (range 1.0 to 8.0). The average length of post-operative follow-up was 21 month (range 2.0 to 60).

**Table 1:** Baseline patient characteristics

	Average age of referral (years old)	Male to female ratio (M:F)	Percentage of patients with family history of strabismus	Average spherical equivalence (D)	Average age of esotropia onset (years old)	Average age of starting spectacle correction (years old)	Average time between onset of esotropia to spectacle correction (month)	Average time between spectacle correction to surgery (month)	Average age of surgery (years old)
Baseline patient characteristics	2.85 (0.50–8.0)	23:24	24/47 (53.2%)	+ 6.03 (+ 4.00 to + 9.8)	1.34 (0–3.75)	1.88 (0.25–4.0)	7.17 (0–36)	21.8 (2–80)	3.62 (1–8)

### Pre-operative alignment in primary gaze and sensory fusion status

Pre-operatively, the average amount of distance eso-deviation without spectacle correction was 39 PD (range 30 to 55 PD). The average amount of distance eso-deviation with hyperopic spectacle correction was 26 PD (range 0 to 45 PD). The average amount of near eso-deviation without spectacle correction was 45 PD (range 30 to 55 PD). The average amount of near eso-deviation with hyperopic spectacle correction was 32 PD (range 14 to 50 PD). The average amount of near eso-deviation with hyperopic spectacle with + 3.0 reading addition was 22.1 PD (range 4 to 40 PD).

Pre-operatively, 2 of the 47 (4.3%) patients were able to demonstrate sensory fusion by demonstrating peripheral fusion on Worth 4-dot testing and/or documented stereopsis.

### Post-operative alignment in primary gaze

At the last post-operative visit, the average amount of distance eso-deviation with hyperopic spectacle correction was 2.3 PD (range − 16 to 20 PD). The average near eso-deviation with hyperopic spectacle correction was 4.7 PD (range − 20 PD to 30 PD). Comparison of pre- and post-operative ocular alignment is listed in Table 2.

**Table 2:** Comparison of pre-operative and post-operative ocular alignment

	Distance deviation with spectacle correction (PD)	Near deviation with spectacle correction (PD)	Near deviation with spectacle correction and + 3.0 reader (PD)
Pre-operative measurement	ET 25.5 (Ortho to ET 45)	ET 32.3 (ET 14 to ET 50)	ET 22.1 (ET 4 to ET 40)
Post-operative measurement	ET 2.32 (XT 16 to ET 20)	ET 4.72 (XT 20 to ET 30)	ET 4.86 (XT 2 to ET 12)

### Surgical success and post-operative sensory fusion status

Post-operatively, 35 patients (74.5%) achieved surgical success, 4 patients (8.5%) were over-corrected and 8 patients (17.0%) were under-corrected. For the 4 patients who were over-corrected and presented with consecutive exotropia larger than 10 PD, 2 were managed by reducing their hyperopic spectacle correction and 2 required later bilateral medical rectus advancement. For the 8 patients who had residual esotropia of more than 10 PD, 1 required additional surgery to correct the residual esotropia and the other 7 patients were managed without further surgical intervention. Re-operation was not offered to undercorrected patients unless their residual eso-deviation was greater than 15 PD or if patients/parents declined more surgery. A total of 3 patients (6.38%) required additional strabismus surgery after the initial bilateral medial recession surgery.

**Fig. 1:**
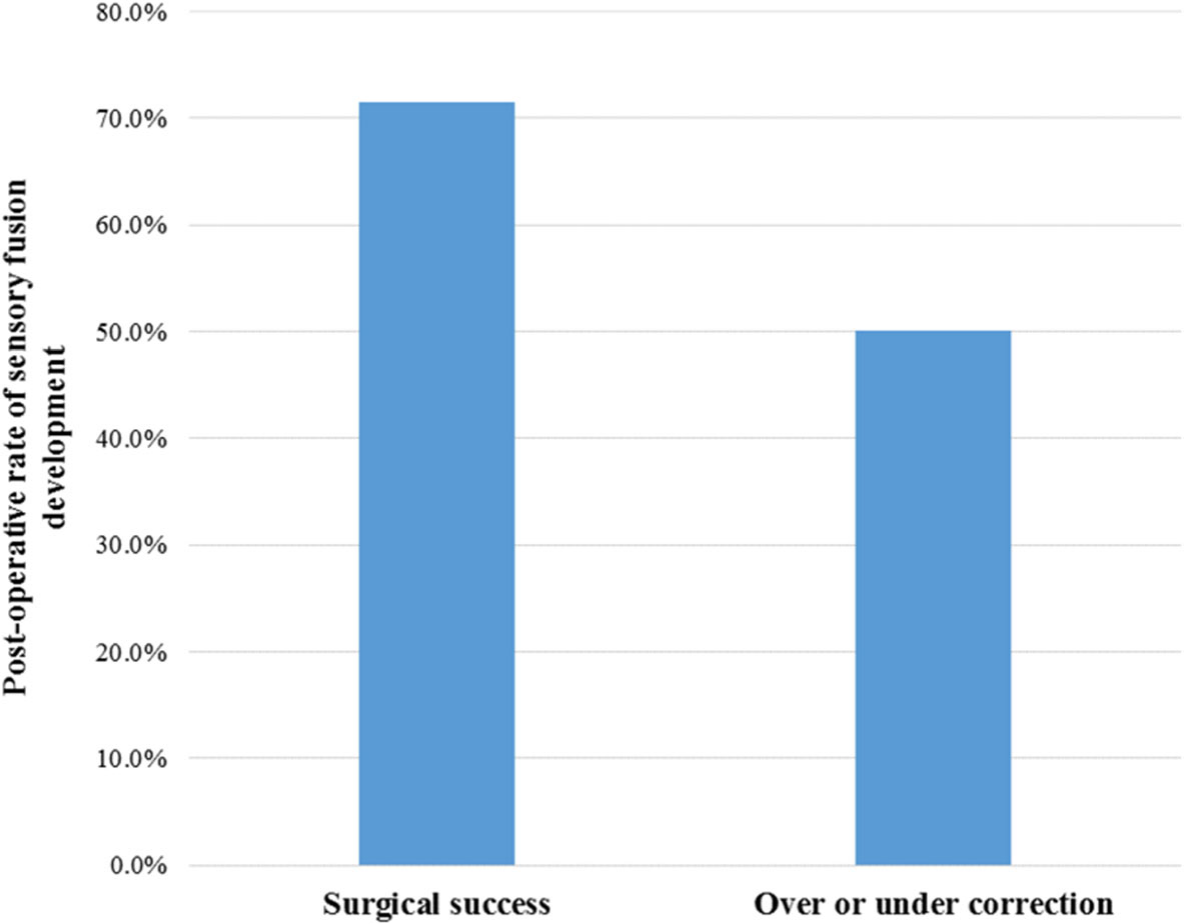
Rate of sensory fusion development and post-operative ocular alignment. For patients who achieved ocular alignment within 10 PD of orthotropia, 71.4% demonstrated sensory fusion, compared with 50.0% for patients who were either over- or under-corrected (*P* = 0.18)

**Fig. 2:**
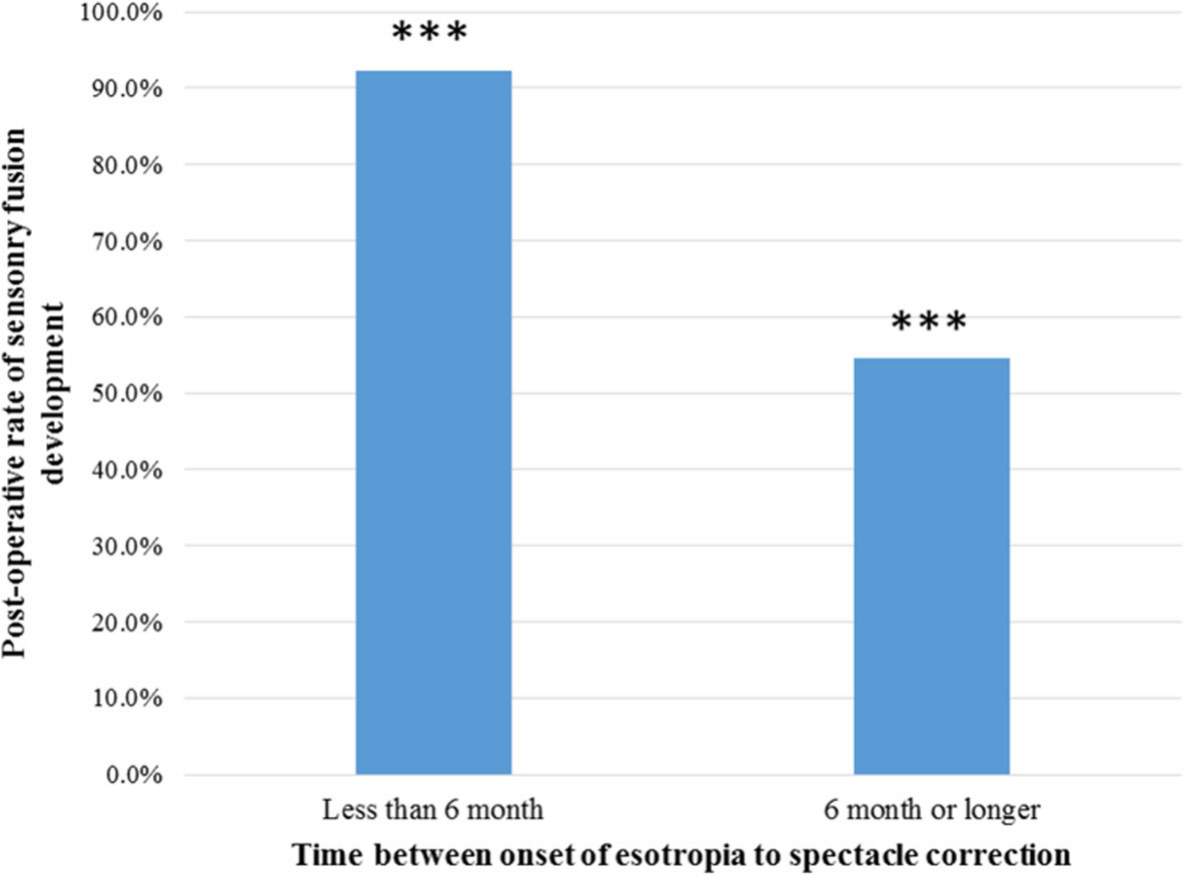
Rate of sensory fusion development and time interval between the onset of esotropia to spectacle correction. For patients who received hyperopic spectacle correction within 6 month of esotropia onset, 92.3% demonstrated sensory fusion, compared at 54.5% for patients who received hyperopic spectacle correction 6 month or longer after the onset of esotropia (P = 0.02). *** indicates statistical significance

Overall, 31 patients (66%) demonstrated sensory fusion with either binocular single vision (BSV) on Worth 4-dot testing and/or measured stereopsis at their last follow up appointment. For patients who were considered surgical success, 71% (25 out of 35 patients) demonstrated sensory fusion, compared at 50% (6 out of 12 patients) for patients who were either over- or under-corrected (*P* = 0.18) (Fig. 1). For patients who received hyperopic spectacle correction within 6 months of esotropia onset, 92% (12 out of 13 patients) demonstrated sensory fusion. For patients who received hyperopic spectacle correction 6 months or longer after the onset of esotropia, 55% (13 out of 33 patients) demonstrated sensory fusion (*P* = 0.02) (Fig. 2). For patients who received BMR surgery within 12 months of using hyperopic spectacle correction, 50% (7 out of 14 patients) demonstrated sensory fusion, compared at 72% (23 out of 32 patients) for patient who received BMR surgery at 12 months or later after starting hyperopic spectacles (*P* = 0.16).

## Discussion

For patients with esotropia associated with high hypermetropia, the use of augmented surgery and/or prism adaptation have allowed for significantly improved surgical outcomes than traditional surgery focusing only on the non-accommodative component after spectacle correction [4, 6–9]. In 1993, Wright and Bruce-Lyle introduced augmented surgery for children with esotropia associated with high hypermetropia based on the average of near deviation with and without spectacle correction with high rate of surgical success [8]. Since then, Hwang et al. have demonstrated similar motor and sensory surgical outcomes for patients who were managed with either augmented surgery or pre-operative prism adaptation based surgery [10]. In our study, our surgery planning was performed as per the nomogram table for the near angle as the surgical angle [11]. Pre-operatively, prism adaptation testing was added to the pre-operative surgical planning, if post-operative diplopia testing with loose prisms for distance continued to demonstrate diplopia. This is essentially similar to the augmentation strategy based on the average of near deviation with and without spectacle correction [8].

Although lower than the 88% rate of ocular alignment success that Wright and Bruce-Lyle reported, we achieved an excellent rate of alignment success (within 10 PD of orthotropia) of 75%, with an under-correction rate of 17%. Of the 4 patients (8.5%) who showed a consecutive exotropia of more than 10 PD, half were successfully managed with reducing hyperopic spectacle correction and half required re-operation. One of the possible reasons for the lower rate of surgical success in our study is that our study included patients with a much higher degree of hypermetropia with an average spherical equivalence of more than + 6.0 D, compared with previous studies [5, 8, 10]. The other important consideration for patients with high hypermetropia is the effect of high plus spectacles on the measurement of strabismus, as the true deviation will be greater than the measured deviation with plus spectacles when measured with standard tables [12, 13]. For patients with high plus spectacles, surgeries based on measured deviation may result in under-correction.

Overall, excellent post-operative alignment was achieved for patients with high hypermetropia in the study, as the average post-operative distance esotropia with spectacle correction was only 2.3 PD. However, the large range of post-operative alignment from exotropia of 16 PD to esotropia of 20 PD demonstrates the high variabilities can be seen post-operatively in this group of patients, which has been previously reported in the literature [5]. Although our study showed a low rate of re-operation at 6.4% compared to the 37% rate of re-operation reported by Arnoldi, it is still crucial for surgeons to thoroughly discuss and highlight the risk of needing additional surgeries during the consenting process for children with high hypermetropia [5].

Pre-operatively, only 4.3% of patients were able to demonstrate sensory fusion, this is likely the result of the large angle esotropia prior to surgery, as well as the young age of patients at pre-operative visits when sensory fusion demonstration is difficult and less reliable. Nevertheless, the percentage of patients able to demonstrate sensory fusion increased dramatically after surgery, from 4.3 to 66%. Although not statistically significant, we found a trend toward better rate of sensory fusion for patients who achieved surgical success (71%), compared to patients who were either over- or under-corrected (50%) (Fig. 1). It is interesting to point out that patients who were either over- or under-corrected achieved relatively high rate of sensory fusion. This is likely because that the majority of the over- or under-corrected patients have a better chance at achieving sensory fusion with the reduced amount of deviation post-operatively. Furthermore, management options such as reducing hyperopic spectacle correction for over-corrected patients and additional strabismus surgeries are always available for over- or under-corrected patients.

In our study, the timing between the onset of esotropia and the start of spectacle correction based on cycloplegic refraction was found to be a key factor in determining the functional outcomes for patients with high hypermetropia. Patients who received hyperopic spectacle correction within 6 month of the onset of esotropia achieved a higher rate of sensory fusion development than patients who received spectacle correction 6 month or later after the onset of esotropia (*P* = 0.02). In contrast, the timing between starting hyperopic spectacle correction and strabismus surgery was found not to be a factor in the functional outcome in our study (*P* = 0.16), this is in keeping with previous reported studies in the literature [14, 15]. The long average time (21.8 month) between spectacle correction and surgical intervention reported in our study was likely the result of local practice and referral pattern. Often times, patients were prescribed spectacle glasses and managed conservatively by patient’s family physician or optometrist for a considerable amount of time before a referral to a pediatric ophthalmology at a tertiary center takes place.

The findings in this study indicate that prompt ocular assessment with cycloplegic refraction and timely spectacle correction are crucial in the management of patients presenting with esotropia with high hypermetropia, whereas timing of strabismus surgery apparently does not affect the functional outcomes post-operatively and possibly may not be a factor in the management of patients with esotropia with high hypermetropia. This contrasts with infantile esotropia, where early surgical intervention is associated with higher likelihood of developing stereopsis [16].

## Conclusions

In summary, augmented bilateral medial recession for esotropia associated with high hypermetropia has a high rate of surgical success with low rate of under- or over-correction. There is a trend toward higher rate of sensory fusion development for patients who are considered surgical success. Shorter time interval between the onset of esotropia and the use of hyperopic spectacles is found to be associated with better functional outcomes, whereas timing between the use of hyperopic spectacles and strabismus surgery does not seem to influence the rate of sensory fusion development.

## Data Availability

Data and materials are available upon request from the corresponding author at Bo.Li@sjhc.london.on.ca

## Abbreviations

BMR: Bilateral medial recession
BSV: Binocular single vision
D: Diopter
PD: Prism diopter
